# Differential expression of HIF-1α in CD44^+^CD24^-/low ^breast ductal carcinomas

**DOI:** 10.1186/1746-1596-6-73

**Published:** 2011-08-08

**Authors:** João Paulo Oliveira-Costa, Juliana S Zanetti, Giórgia G Silveira, Danilo F Soave, Lucinei R Oliveira, Verônica A Zorgetto, Fernando A Soares, Sérgio Zucoloto, Alfredo Ribeiro-Silva

**Affiliations:** 1 Department of Pathology, Ribeirao Preto Medical School, University of Sao Paulo, Ribeirao Preto, Sao Paulo, Brazil; 2 Department of Pathologic Anatomy, A. C. Camargo Cancer Hospital, Brazil; 3 Department of Pathology, Ribeirão Preto Medical School, University of São Paulo, Avenida Bandeirantes 3900, Bairro Monte Alegre, 14049-900, Ribeirão Preto, São Paulo, Brazil

**Keywords:** breast cancer, CD44, CD24, HIF-1α, hypoxia, immunohistochemistry, prognosis, stem cell

## Abstract

**Background:**

Cancer stem cell (CSC) hypothesis postulates that tumors are maintained by a self-renewing CSC population that is also capable of differentiating into non-self-renewing cell populations that constitute the bulk of tumor. Stem cells renewal and differentiation can be directly influenced by the oxygen levels of determined tissues, probably by the reduction of oxidative DNA damage in hypoxic regions, thus leading to a friendlier microenvironment, regarding to clonal expansion and for resistance to chemotherapeutic regimens. Furthermore, there have been strong data indicating a pivotal role of hypoxic niche in cancer stem cells development. There are evidence that hypoxia could drive the maintenance of CSC, via HIF-1α expression, but it still to be determined whether hypoxia markers are expressed in breast tumors presenting CD44^+^CD24^-/low ^immunophenotype.

**Methods:**

Immunohistochemical analysis of CD44^+^CD24^-/low ^expression and its relationship with hypoxia markers and clinical outcome were evaluated in 253 samples of breast ductal carcinomas. Double-immunolabeling was performed using EnVision Doublestain System (Dako, Carpinteria, CA, USA). Slides were then scanned into high-resolution images using Aperio ScanScope XT and then, visualized in the software Image Scope (Aperio, Vista, CA, USA).

**Results:**

In univariate analysis, CD44^+^CD24^-/low ^expression showed association with death due to breast cancer (p = 0.035). Breast tumors expressing CD44^+^CD24^-/low ^immunophenotype showed relationship with HIF-1α (p = 0.039) and negativity for HER-2 (p = 0.013).

**Conclusion:**

Considering that there are strong evidences that the fraction of a tumour considered to be cancer stem cells is plastic depending upon microenvironmental signals, our findings provide further evidence that hypoxia might be related to the worse prognosis found in CD44+CD24-/low positive breast tumors.

## Background

The cancer stem cell (CSC) hypothesis postulates that tumors are maintained by a self-renewing CSC population that is also capable of differentiating into non-self-renewing cell populations that constitute the bulk of the tumor [1]. The first insight indicating a role of breast cancer stem cells (BCSC) in breast carcinogenesis was demonstrated by the study of Al-Hajj and co-workers, where Lin^-^CD44^+^CD24^-/low ^cells injected into the mammary fat pad of non-obese diabetic/severed combined immunodeficient (NOD/SCID) mice were able to form palpable tumors, even with just a few hundred of cells being transplanted. Another interesting observation in the same study was the fact that Lin^-^CD44^+^CD24^-/low ^cells were not only able to give origin to additional Lin^-^CD44^+^CD24^-/low ^cells, but also generated phenotypically distinct cells, indicating that BCSC could generate an homogeneous population of non-tumorigenic cells, besides the generation of new BCSC [2].

Stem cells renewal and differentiation can be directly influenced by the oxygen levels of determined tissues, probably by the reduction of oxidative DNA damage in hypoxic regions, thus leading to a friendlier microenvironment, regarding to clonal expansion and for resistance to chemotherapeutic regimens [3]. Furthermore, there have been strong data indicating a pivotal role of hypoxic niche in cancer stem cells development, especially in glioblastomas [4,5].

Hypoxia-inducible factor 1 (HIF-1) is a heterodimeric complex consisting of α and β subunits. There are 3 human HIF-α genes (HIF-1α, HIF-2α, and HIF-3α) that are oxygen sensitive, although only the first 2 have been extensively studied. HIF-1α is the best characterized and the most ubiquitously expressed, functioning as a regulator of oxygen homeostasis in many cell types [6]. High levels of HIF-1α have been correlated with tumor progression and poor prognosis in patients with brain, non-small-cell lung, breast, ovarian, uterine, and cervical cancers [7].

Hypoxia-regulated protein carbonic anhydrase IX (CAIX) has been studied in several tumors and its expression has been related with clinical outcome [8-10]. CAIX regulates tissue pH and is induced by hypoxia, mainly in an HIF-1-dependent manner, playing an important role on maintenance of hypoxia state in solid tumors [10]. Nevertheless, the relationship between CAIX expression and CSCs in breast cancer was not addressed.

The relationship between hypoxia markers and CD44^+^CD24^-/low ^immunophenotype in breast tumors still poorly understood. Our study aims to investigate the prognostic impact of CD44^+^CD24^-/low ^immunoexpression in breast cancer. Further, we hope to understand its relationships with hypoxia markers (HIF-1α and CAIX), clinical outcome, clinicopathological findings and immunohistochemical prognostic markers, including estrogen receptor (ER), progesterone receptor (RP), human epidermal growth factor receptor-2 (HER-2), breast cancer gene 1 (BRCA1), the proliferation marker Ki67 and p53.

## Methods

### Patients and clinicopathologic information

A total of 300 cases of invasive ductal carcinoma of the female breast diagnosed between 1994 and 2004 were retrieved from the files of the Department of Pathology of the General Hospital of Ribeirao Preto School of Medicine, University of Sao Paulo, Brazil. From the medical registers, the following information was retrieved: age, menopausal status, tumor size, metastasis to regional lymph nodes, recurrence, distant metastasis, death, disease-specific survival and disease-free survival. Patients who received antiestrogen or radion and/or chemotherapy had been previously excluded from the study. Disease-specific survival (DSS) was defined as the number of months from diagnosis to the time of death due to breast cancer and disease-free survival (DFS) was defined as the time from diagnosis to the occurrence of local recurrence or metastasis.

For each case, each available hematoxylin and eosin (H&E)-stained section was reviewed to confirm the diagnosis of invasive ductal carcinoma and to delimitate a representative block for tissue microarray construction. Cases without complete data and/or sufficient material were excluded from the study. The cases were graded according to the current guidelines of the Scarff-Bloom & Richardson grading system modified by Elston & Ellis [11]. None of patients had received any treatment before the biopsy procedure. The study protocol conformed to the ethical guidelines of the 1975 Declaration of Helsinki and was approved by the local Ethics Committee.

### Tissue microarray (TMA)

The hematoxylin and eosin (H&E) slides were reviewed by two experienced pathologists (ARS and FRV) to delimit the most significant area of each tumor. This region was then selected to the construction of tissue microarray (TMA) paraffin block. For TMA block construction, core biopsies (diameter, 1 mm) were punched from the selected regions of each of the 300 donor paraffin blocks and arrayed into a new recipient paraffin block using a Manual Tissue Arrayer I (Beecher Instruments, Silver Spring, USA). Three-μm-thick sections were cut from TMA paraffin block using the Paraffin tape-transfer system (Instrumedics, Saint Louis, USA). One section was stained with H&E to confirm the presence of the tumor by light microscopy. Immunohistochemistry was carried out on two slides at different depth levels to increase the accuracy and strength of the array-based data.

### Immunohistochemistry

**Figure 1:**
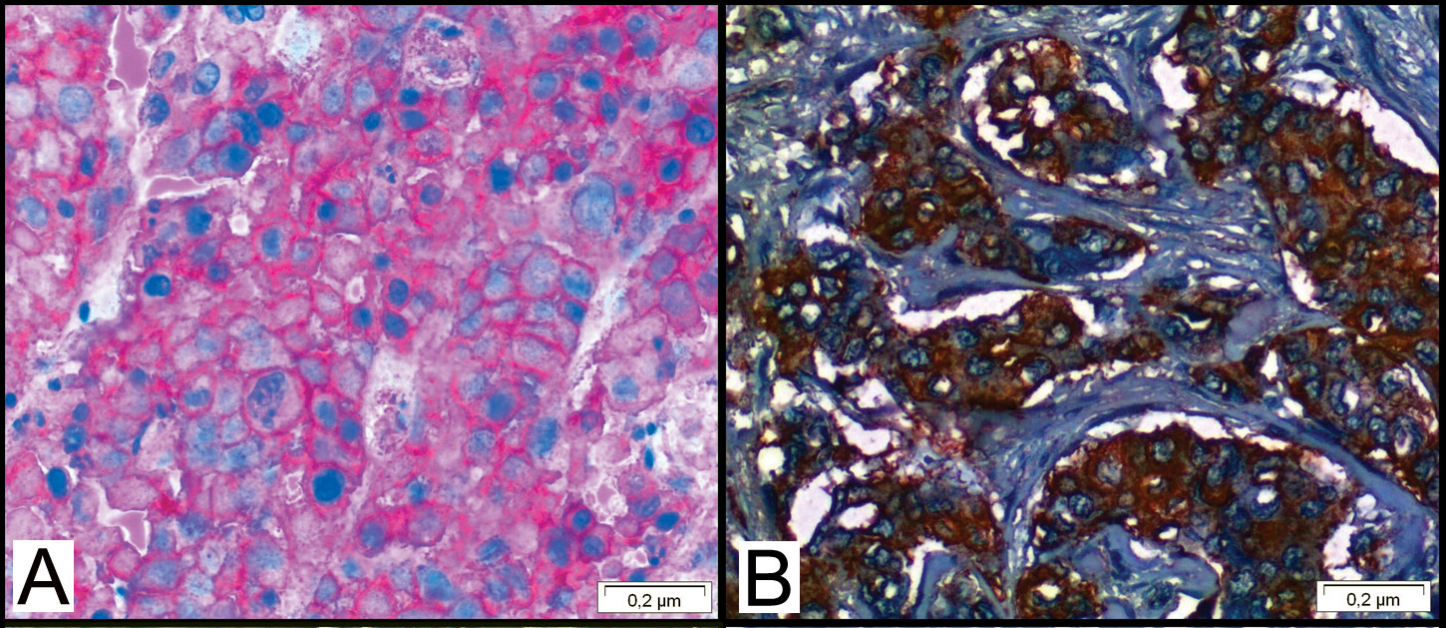
**Double immunostaining showing CD44 (brown) and CD24 (red)**. A) Immunofenotype CD44-CD24+. B) Immunofenotype CD44+CD24+ (for means of comparison).

Tissue samples had been routinely fixed in 4% neutral formalin and embedded in paraffin. Immunohistochemical staining was developed using the Novolink™ Max Polymer Detection System (Novocastra, Newcastle Upon Tyne, UK). Briefly, paraffin sections were de-waxed in xylene and rehydrated through a series of graded alcohols. Endogenous peroxidase activity was blocked for 30 min in a solution containing 0.3% of hydrogen peroxide in order to block non-specific immunoassaying. The sections were then placed in 10 mM citrate buffer and submitted to heat retrieval using a vapor lock for 40 minutes. After antigen retrieval, specimens were allowed to cool for 30 minutes at room temperature, and then incubated at 4°C overnight with primary antibody. The dilution and source of the primary antibodies used in this study were: CD44 (1:50, clone DF1485, Novocastra, Newcastle upon Tyne, UK), CD24 (1:50, clone SN3b, Thermo Scientific, Fremont, CA, USA), HIF-1α (1:50, monoclonal, Abcam, Cambridge, UK), CAIX (1:100), p53 (1:50, clone DO-7, Novocastra, Newcastle upon Tyne, UK), estrogen receptor (ER) (1:100 clone 6F11, Novocastra, Newcastle upon Tyne, UK), progesterone receptor (PR) (1:100, clone 16, Novocastra, Newcastle upon Tyne, UK), and c-erbB-2 (1:100, clone CB11, Novocastra, Newcastle upon Tyne). After the overnight incubation with the primary antibody, the slides were incubated with post primary solution for 30 minutes and then incubated with the polymer for 30 minutes (both provided by the Novolink™ Max Polymer Detection System). The reaction was stained with diaminobenzidine (DAB) followed by hematoxylin counterstaining. The slides were then dehydrated in an ethanol series and mounted with Permount (Fischer, Fairlawn, NJ). Double-immunolabeling was performed according to the manufacturer's instructions (EnVision Doublestain System, Dako, Carpinteria, CA, USA). CD44 was stained with DAB (brown), and CD24 was stained with fast red (red) (Figure 1)

Glioblastoma cases were used as positive control for HIF-1α. Cases of invasive ductal carcinoma previously known to be positive for CD24, CAIX, ER, PR, p53, and HER-2 were used as positive controls for those markers. Negative controls for immunostaining were prepared by omission of the primary antibody.

### Image analysis

CD44 was identified as brown and CD24 as red membrane staining. Cells with brown color staining without much interference from red color were identified as CD44+/CD24-/low tumor cells, whereas cells with intense red staining in the absence of brown color were characterized as CD44-/CD24+ tumor cells. The slides were then scanned into high-resolution images using Aperio ScanScope XT (Aperio, Vista, CA, USA). The images were then visualized in the software Image Scope (Aperio, Vista, CA, USA).

To define the cut-off point for CD44 and CD24 we have followed the criteria of Giatromanolaki and collaborators [12]. CD44 and CD24 positive cases were considered as those which had more than 10% of stained membranes. For HIF-1α and CAIX we followed the criteria of Chen et al [13]. Were considered positive to HIF-1α cases with more than 1% of nuclear staining and, to CAIX, cases with more than 1% of membranous staining. The cases in which more than 10% of neoplastic cells stained for Ki67 (nuclear staining) were considered highly proliferative. The cases were interpreted as ER, PR, BRCA1 and p53 positive if more than 10% of the neoplastic cells showed nuclear staining [11].

HER-2 immunoexpression was scored according to current guidelines [15]. For statistical purposes, carcinomas with score 0 or 1+ were considered negative; and carcinomas with score 3+ were considered positive. Cases with HER-2 scored as 2+ were submitted to chromogenic in situ hybridization (CISH) for HER2.

All immunostains and CISH slides were independently analyzed and scored by two experienced pathologists (ARS and FRV). Whenever a discrepancy between the readings occurred, the two pathologists discussed the case in a multi-head microscope and reached a consensus agreement for the final scoring.

### Chromogenic in situ hybridization (CISH)

Sections (3 μm) were cut from the original paraffin blocks in the cases in which the HER2 was graded as 2+ by the immunohistochemical method. The ZytoDot 2C SPEC HER2/CEN 17 probe kit (Zytovision, Bremerhaven, Germany) was used for the detection of the human HER2 gene and alpha-satellites of chromosome 17. All procedures were performed step by step according to the manufacturer's instructions. Using this kit, two green (HER2) and two red (CEN 17) signals are expected in a normal interphase nucleus. HER2 was considered amplified when the HER2/CEN 17 ratio was ≥ 2 on average for 60 cells [14]. Only the cases scored as 2+ by the immunohistochemical method in which the HER2 was amplified on CISH analysis were considered positive.

### Statistical analysis

SPSS Statistics 18.0 (Chicago, IL, USA) was used to perform statistical analyses and tests. The relationships between CD44^+^CD24^-/low ^expression, immunohistochemical findings and clinicopathological features were tested with cross tables applying the χ^2 ^(three or more variables) or Fisher tests (two variables), and all tests were 2-tailed. The Kaplan-Meier curves using the log-rank test were used to estimate and compare disease-specific survival, disease-free survival and metastasis-free survival. In order to observe the independent prognostic value of the variables we performed a Cox Proportional Hazards Model. A p-value of < 0.05 was considered significant.

## Results

### Demographics of the patients and tumor characteristics

Of the 300 cases initially selected to this study 253 had all clinical data and sufficient material for tissue microarray construction and immunohistochemistry analysis.

The relationship between CD44^+^CD24^-/low ^immunophenotype expression and clinicopathological findings is summarized in Table 1. The age of the patients ranged between 19 to 91 years (mean, 56,19 years). All patients were female. Ninety two patients (35%) were premenopausal and 171 were post-menopausal (65%). All tumors were classified as invasive ductal carcinomas (ICDs), with sizes ranging from 1 to 18 cm (median, 4.45 cm). The tumors were classified as grade 1 (97 [36.9%]), grade 2 (126 [47.9%]) and grade 3 (40 [15.2%]). Seventy three patients (28.9%) had tumors with less than 2 cm, 105 had tumors between 2 and 5 cm (41.5%) and 75 had tumors larger than 5 cm (29.6%). One hundred and twenty three (46.8%) patients had no metastatic lymph nodes, while 140 (53.2%) were lymph node positive. According to the Clinical Staging System, created by the American Joint Committee on Cancer (Singletary et al., 2003), thirty five (13.3%) were classified as stage I, 64 (24.3%) were IIa, 55 (20.9%) were IIb, 27 (10.3%) were IIIa, 66 were IIIb (25.1%), and 16 were IV (6.1%). Distant metastasis occurred in 81 (30.8%) patients and 178 (67.7%) died due to breast cancer.

**Table 1:** Relationship between clinical and pathological features and breast carcinomas expressing the immunophenotype CD44^+^CD24^-/low ^and carcinomas that do not express this immunophenotype

Feature	CD44^+^CD24^-/low ^positive	CD44^+^CD24^-/low ^negative	p-value
Age (years)			0.789

< 50	38	66	

> 50	51	98	

			

Menopausal status			0.131

Pre	38	53	

Post	51	111	

			

Tumor size (cm)			0.837

< 2	24	49	

2-5	39	66	

> 5	26	49	

			

Bloom & Richardson			0.319

I	28	66	

II	48	73	

III	13	25	

			

Clinical Staging			0.219

I	12	21	

IIa	21	40	

IIb	19	34	

IIIa	6	20	

IIIb	22	44	

IV	9	5	

			

Recurrence			1.000

Yes	11	21	

No	78	143	

			

Lymph node status			0.895

Pos	47	89	

Neg	42	75	

			

Distant metastasis			0.479

Yes	30	48	

No	59	116	

			

Death			0.035*

Yes	37	46	

No	52	118	


*Statistically significant.

CD44^+^CD24^-/low ^expression showed association with death due to breast cancer (p = 0.035) but not with age (p = 0.789), menopausal status (p = 0.131), clinical stage (p = 0.219), histological grade (p = 0.319), lymph node status (p = 0.895), recurrence (p = 1.000), or distant metastasis (p = 0.479).

### Immunohistochemical results

The relationship between CD44^+^CD24^-/low ^immunophenotype expression and immunohistochemical findings is summarized in Table 2. In our study, we found a CD44^+^CD24^-/low ^phenotype in 92 of 253 cases (35%) (Figure 1). The positivity rate for ER, PR, HER-2, and p53 were, respectively, 63.6% (161 cases), 59.3% (150 cases), 19.8% (50 cases) and 64% (162 cases). The positivity rate for the hypoxia markers used in this study was 55 cases (21.7%) for HIF-1α, 67 cases (26.5%) for CAIX immunoexpression.

**Table 2:** Relationship between immunohistochemical features and breast carcinomas expressing CD44^+^CD24^-/low ^immunophenotype and carcinomas that do not express this immunophenotype

Feature	CD44^+^CD24^-/low ^positive	CD44^+^CD24^-/low ^negative	p-value
RE			0.340

Pos	53	108	

Neg	36	56	

			

RP			0.141

Pos	47	103	

Neg	42	61	

			

p53			0.786

Pos	56	106	

Neg	33	58	

			

HER-2			0.013*

Pos	10	40	

Neg	79	124	

			

Ki67			0.410

Pos	34	54	

Neg	55	110	

			

HIF-1a			0.039*

Pos	26	29	

Neg	63	135	

			

CAIX			0.551

Pos	26	41	

Neg	63	123	

*Statistically significant.

CD44^+^CD24^-/low ^immunophenotype showed relationships with HIF-1α (p = 0.039) and negativity for HER-2 (p = 0.013). There were no relationships with the expression levels of estrogen (p = 0.340) and progesterone (p = 0.141) receptors, Ki67 (p = 0.410), CAIX (p = 0.551) and p53 (p = 0.786) (Table 1).

### CD44^+^CD24^-/low ^immunophenotype and survival

**Figure 2:**
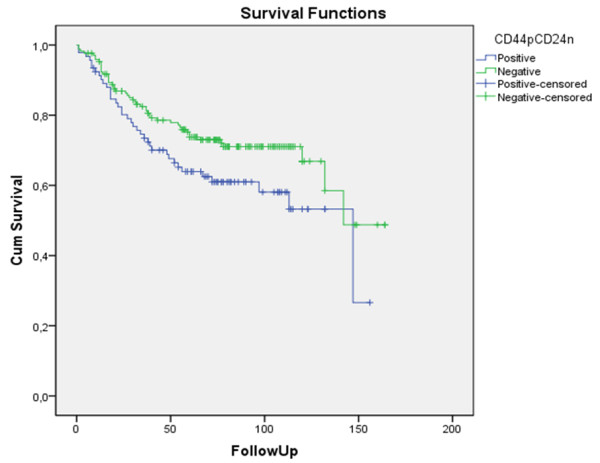
**Univariate analysis of the prognostic impact of CD44^+^CD24^-/low ^status on disease-specific survival of breast cancer patients**. Using Kaplan Mayer table followed by log-rank test.

**Figure 3:**
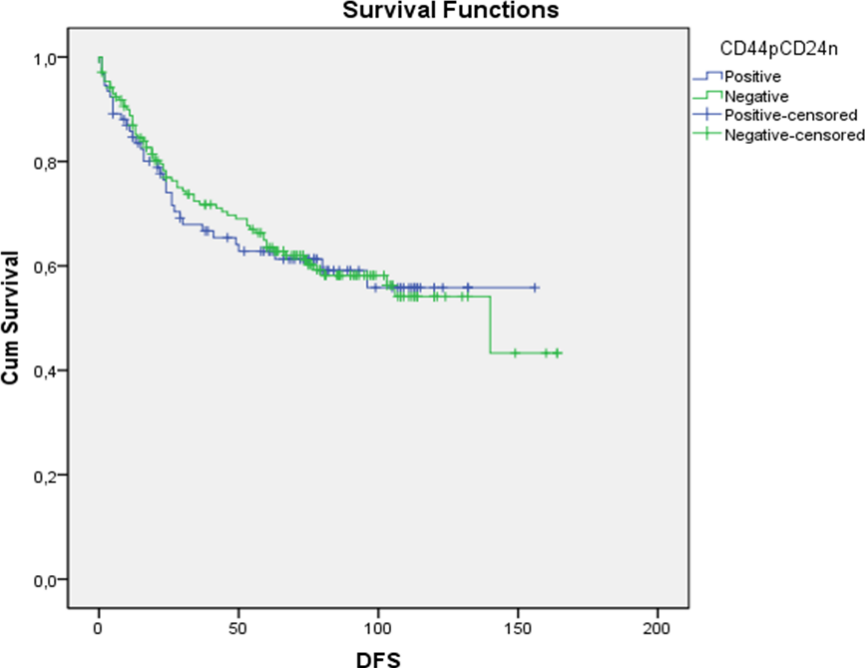
**Univariate analysis of the prognostic impact of CD44^+^CD24^-/low ^status on disease-free survival of breast cancer patients**. Using Kaplan Mayer table followed by log-rank test.

The follow up period for the patients in the study ranged from 1 to 164 months, and the mean survival time was 66.96 months. Using Kaplan-Meier survival analysis with the log-rank test, we found significantly worse disease-specific survival (p = 0.035) for the CD44^+^CD24^-/low ^cases (Figure 2). No differences were found in disease-free survival (p = 0.720) (Figure 3). In multivariate analysis, CD44^+^CD24^-/low ^expression was not an independent prognostic factor.

## Discussion

In this study, we determined the relationships between CD44^+^CD24^-/low ^phenotype and hypoxia markers, HIF-1α and CAIX, and classical prognostic factors in breast pathology, including clinicopathological features and the expression of immunohistochemical markers of prognostic significance (HER2 status, p53 expression and hormonal status). To the best of our knowledge this is the first study demonstrating an association between hypoxia markers and the CD44^+^CD24^-/low ^phenotype in a large series of breast invasive ductal carcinomas, by immunohistochemistry.

Similar to the results of Honeth and co-workers, we found 35% of cases showing CD44^+^CD24^-/low ^immunophenotype, suggesting that not all breast tumors contain cells with this phenotype, as described by Al-Hajj in 2003 [16,2].

Since the discovery of the high capacity of generating new tumor cells by mammary CD44^+^CD24^-/low ^cells, this phenotype are associated with worse survival and aggressive behavior [17]. Sheridan and co-workers showed that CD44^+^CD24^-/low ^phenotype is associated with enhanced invasive properties and elevated expression of genes involved in invasion [18]. Further, the CD44^+^CD24^-/low ^cell population was related with poor prognosis, although not in all studies [12]. In the study of Mylona and cols., the CD44^+^CD24^-/low ^phenotype was emerged as a poor prognostic indicator, within the group of grade II tumors [19]. Furthermore, clinical studies indicate that CD44+/CD24- tumor-initiating cells express an invasive gene signature and may be associated with distant metastases [20-22]. Although the mechanisms of this aggressive behavior are not fully understood, it is now accepted that the generation of different subclones provides tools to tumorigenic cells proliferation and invasion abilities, leading to metastasis and insensitivity to classical treatments [17,23].

Here we demonstrated that CD44^+^CD24^-/low ^breast tumors show an association with HIF-1α status, but not with CAIX. Recent studies have shed light in the relationship between cancer stem cells and tumor hypoxia. There are strong evidences that the fraction of a tumour considered to be cancer stem cells is plastic depending upon microenvironmental signals such as hypoxia [24]. Soeda and collaborators demonstrated that culture in hypoxia and activation of HIF-1α expands the sub-population of cells positive for markers associated with a more stem-like phenotype, such as CD133 and CD44 (25). A study from Pahlman and colleagues has also shown that hypoxia in neuroblastoma cells can alter gene expression to that of a more immature phenotype.^26 ^There are recently published data demonstrating this critical role of hypoxia in maintaining the stem-like fraction of cancer cells [27,4,28].

Low oxygen levels promote maintenance of embryonic stem cell pluripotent potential and block differentiation [29]. In a recent study, Bar and co-workers showed that gliobastoma neurosphere cell lines and freshly resected tumors respond to hypoxia by an increase in cancer stem cell marker CD133 expression. These neurospheres also exhibited a significant increase in clonogenic capacity, after 12 and 24 hours of hypoxia. According to the authors, HIF-1α alone can mediate the majority of hypoxic effects in gliobastoma neurospheres, indicating a pivotal role of HIF-1α in clonal expansion of cancer stem cells [5].

Further findings indicate that more than co-acting in cancer stem cell maintenance, hypoxia could drive the maintenance of a cancer stem cell niche, with a strong participation of HIF-1α. Heddleston and colleagues showed that non-stem glioma cells cultured under hypoxic conditions had twice the rate of formation of neurospheres, compared to cells in normoxia, suggesting that a hypoxic microenvironment plays a pivotal role in promoting and maintaining the ability of self-renew in stem-like cells, and even conferring self-renewal capability to non-stem cell population [4].

Our finding that HIF-1α is related to CD44^+^CD24^-/low ^immunphenotype in breast ductal carcinomas could indicate that a maintained hypoxia state in breast cancer could facilitate the maintenance of cancer stem cells in these tumors, corroborating the finding that CD44^+^CD24^-/low ^positive tumors have a worse prognosis when compared to non- CD44^+^CD24^-/low ^tumors. The association between negativity to CD44^+^CD24^-/low ^immunophenotype and negativity to HIF-1α and CAIX expression could, at least in part, explain the better survival of this group of patients, and also the worst prognosis associates with CD44^+^CD24^-/low ^cases.

We also found an association between CD44^+^CD24^-/low ^phenotype and HER-2 status. Patients lacking CD44^+^CD24^-/low ^phenotype showed negativity to HER-2 immunohistochemycal expression. Recently, Oliveras-Ferraros and co-workers had demonstrated that CD44^+^CD24^-/low ^cells could be important in de novo resistance to direct HER inhibitors such as trastuzumab (Herceptin^©^), a HER-2 directed antibody, and other studies suggest that the clinical efficacy of trastuzumab may relate to its ability to directly target the CSC population in HER-2-amplified tumors [30,22,23].

In fact HER-2 positive tumors constitute a group with a worse prognosis, when compared to tumors lacking HER-2 expression. The relationship between non- CD44^+^CD24^-/low ^tumors and negativity to HER-2 could explain the better prognosis found in this group of patients.

## Conclusion

The differential expression of hypoxia marker HIF-1α in CD44^+^CD24^-/low ^tumors is in accordance with the current knowledge regarding relationship between microenvironmental factor such as hypoxia or anoxia states with maintenance and propagation of cancer stem cells. Taken together with the differential expression of HER-2, these findings provides further evidence on the worse prognosis found in CD44^+^CD24^-/low ^positive tumors, and might contribute to the more aggressive behavior of these neoplasms. Furthermore, the relationship between hypoxia markers in tumors presenting CSC niche needs to be better understood, and further studies are needed to clarify the molecular basis of these hypoxic CSC-tumors.

## Competing interests

The authors declare that they have no competing interests.

## Authors' contributions

JPOC: concepts, definition of intellectual content and manuscript preparation; JSZ, GGS and DFS: data acquisition and analysis; LRO: design; VAZ: manuscript review; FAS and SZ: performed histological examinations; ARS: study coordination and manuscript review. All authors read and approved the final manuscript.
